# Social Isolation and Sleep Quality of Older Adults in China: Do Family and Friendship Isolation Differ?

**DOI:** 10.1093/geroni/igab046.2197

**Published:** 2021-12-17

**Authors:** Dan Zhang, Zhiyong Lin, Feinian Chen, Shuzhuo Li

**Affiliations:** 1 Xi'an Jiaotong University, Xi'an, Shaanxi, China (People's Republic); 2 The University of Texas at Austin, Austin, Texas, United States; 3 University of Maryland College Park, University of Maryland, Maryland, United States; 4 Xi'an Jiaotong University, Xi’an, Shaanxi, China (People's Republic)

## Abstract

This study provides one of the first population-based studies investigating associations between social isolation, especially its two sub-dimensions (family isolation and friendship isolation), and sleep quality among older adults in China. We address three major research questions: 1) Does the risk of poor sleep quality vary by social isolation status? 2) Are the associations between social isolation and sleep quality mediated by mental disorders (depressive symptoms and loneliness) and physical impairments (pain and comorbidity)? and 3) Does the isolation from family members and friends differ in explaining sleep quality? We analyzed data from the 2014 wave of the China Longitudinal Aging Social Survey (CLASS), in which 7,597 respondents (aged 60-98) had complete information on measures of sleep quality (self-rated sleep difficulty), social isolation (using the Lubben Social Network Scale), and other analytical variables. Logistic regression models were estimated to predict the risk of sleep difficulty and Karlson-Holm-Breen (KHB) decomposition method was employed to test potential mediating effects. Results showed that social isolation, both family and friendship isolation, was significantly associated with higher risks of having sleep difficulty. The adverse effect of family isolation was found to be stronger than that of friendship isolation. Although both mental disorders and physical impairments mediated significant shares of associations between social isolation and sleep quality, physical impairments explained a lesser extent of them than mental disorders. These findings will be helpful for health policymakers and practitioners to design effective intervention strategies to help older adults with sleep problems.

